# Supplementary material for: Epitope Variation in Hemagglutinin and Antibody Responses to Successive A/Victoria A(H1N1) Strains in Young and Older Adults Following Seasonal Influenza Vaccination: A Pilot Study
Source: Vaccines (Basel). 2025 Jul 21;13(7):774. doi: 10.3390/vaccines13070774 (PMC12298324; doi:10.3390/vaccines13070774)
Supplement: Supplementary file 1 [file vaccines-13-00774-s001.zip › vaccines-3681003-supplementary.pdf]

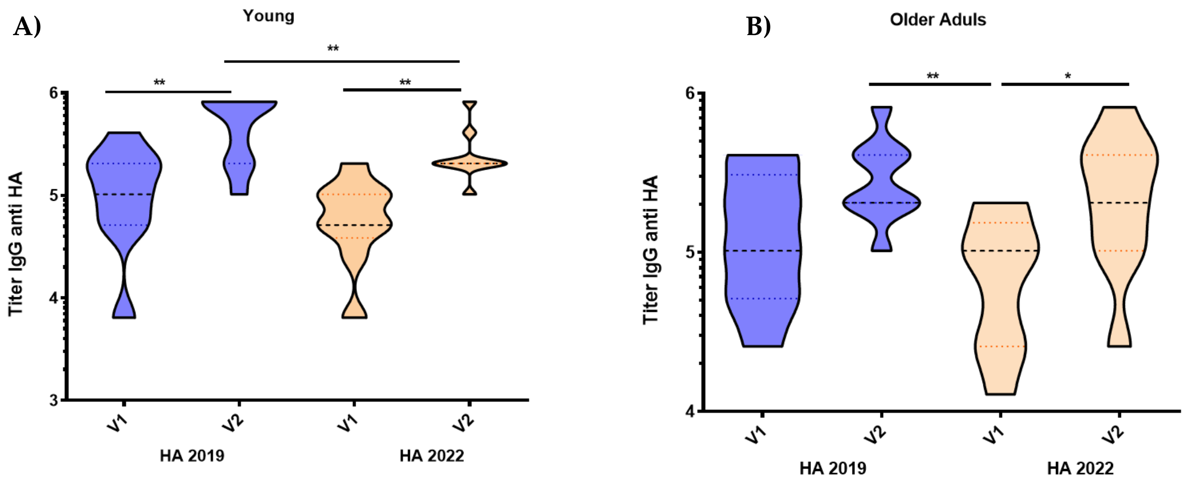

Figure S1. IgG titers against hemagglutinin (HA) from the 2019 and 2022 influenza strains in young individuals (A) and older adults (B), before (V1) and 28 days after (V2) vaccination. Violin plots represent the distribution of the data, with the dashed line indicating the media and dotted lines indicating the quartiles. Statistically significant differences are indicated by asterisks (* $p < 0.05$, ** $p < 0.01$).
